# Machine Learning Models for Predicting Mortality in Patients with Cirrhosis and Acute Upper Gastrointestinal Bleeding at an Emergency Department: A Retrospective Cohort Study

**DOI:** 10.3390/diagnostics14171919

**Published:** 2024-08-30

**Authors:** Shih-Chien Tsai, Ching-Heng Lin, Cheng-C. J. Chu, Hsiang-Yun Lo, Chip-Jin Ng, Chun-Chuan Hsu, Shou-Yen Chen

**Affiliations:** 1 Department of Emergency Medicine, Chang Gung Memorial Hospital, Chang Gung University, Linkou, Taoyuan 333, Taiwan; shihchien0908@gmail.com (S.-C.T.); hsiangyunlo@gmail.com (H.-Y.L.); ngowl@ms3.hinet.net (C.-J.N.); 2 Center for Artificial Intelligence in Medicine, Chang Gung Memorial Hospital, Linkou, Taoyuan 333, Taiwan; chingheng113@gmail.com (C.-H.L.); ccjames@cgmh.org.tw (C.-C.J.C.); 3 Bachelor Program in Artificial Intelligence, Chang Gung University, Taoyuan 333, Taiwan; 4 Graduate Institute of Management, College of Management, Chang Gung University, Taoyuan 333, Taiwan

**Keywords:** machine learning, cirrhosis, gastrointestinal bleeding, emergency department, mortality

## Abstract

Background: Cirrhosis is a major global cause of mortality, and upper gastrointestinal (GI) bleeding significantly increases the mortality risk in these patients. Although scoring systems such as the Child–Pugh score and the Model for End-stage Liver Disease evaluate the severity of cirrhosis, none of these systems specifically target the risk of mortality in patients with upper GI bleeding. In this study, we constructed machine learning (ML) models for predicting mortality in patients with cirrhosis and upper GI bleeding, particularly in emergency settings, to achieve early intervention and improve outcomes. Methods: In this retrospective study, we analyzed the electronic health records of adult patients with cirrhosis who presented at an emergency department (ED) with GI bleeding between 2001 and 2019. Data were divided into training and testing sets at a ratio of 90:10. The ability of three ML models—a linear regression model, an XGBoost (XGB) model, and a three-layer neural network model—to predict mortality in the patients was evaluated. Results: A total of 16,025 patients with cirrhosis and 32,826 ED visits for upper GI bleeding were included in the study. The in-hospital and ED mortality rates were 11.2% and 2.2%, respectively. The XGB model exhibited the highest performance in predicting both in-hospital and ED mortality (area under the receiver operating characteristic curve: 0.866 and 0.861, respectively). International normalized ratio, renal function, red blood cell distribution width, age, and white blood cell count were the strongest predictors in all the ML models. The median ED length of stay for the ED mortality group was 17.54 h (7.16–40.01 h). Conclusions: ML models can be used to predict mortality in patients with cirrhosis and upper GI bleeding. Of the three models, the XGB model exhibits the highest performance. Further research is required to determine the actual efficacy of our ML models in clinical settings.

## 1. Introduction

Cirrhosis is a major cause of morbidity and mortality worldwide [1]. In Asia, in addition to nonalcoholic fatty liver disease and alcoholic hepatitis, viral hepatitis remains a major concern [2]. Viral hepatitis, which is widespread in Taiwan, is the primary cause of liver disease and mortality in this region [3]. Patients with cirrhosis often develop many complications. Among these complications, upper gastrointestinal (GI) bleeding is generally the cause of intubation, complications, and death. Variceal bleeding accounts for 60%–65% of all bleeding episodes in patients with cirrhosis [4]. In individuals with and without cirrhosis, upper GI bleeding is associated with a mortality rate of 23.5% and 11.2%, respectively [5]. In a previous study, we reported that the rate of mortality in patients with cirrhosis and upper GI bleeding was 16% at our hospital [6]. In a study conducted in the United States, Farooq et al. reported that the mortality rate of upper GI bleeding was 5.42% in patients with variceal bleeding and 3.79% in patients with nonvariceal bleeding [7].

Multiple scoring systems are available for evaluating the severity of cirrhosis or predicting the survival of patients with cirrhosis, such as the Child–Pugh score and Model for End-stage Liver Disease (MELD) [8]. However, no scoring systems have yet been developed to evaluate the risk of mortality due to upper GI bleeding in patients with cirrhosis. Machine learning (ML) is being increasingly used in various fields, particularly in the medical field [9,10]. Compared with traditional analysis, ML enables the analysis of larger amounts of information and more accurately identifies potentially valuable items.

In this study, we developed a ML model that can accurately predict mortality in patients with cirrhosis and upper GI bleeding, particularly in emergency department (ED) settings. This model can be used in clinical settings to help clinicians treat high-risk patients earlier and reduce the likelihood of adverse events.

## 2. Materials and Methods

### 2.1. Study Design

In this retrospective study, we analyzed patients’ electronic health records (EHRs) obtained from Chang Gung Memorial Hospital (CGMH), which is currently the largest healthcare institution in Taiwan and consists of seven medical facilities. We focused on patients with a history of cirrhosis who presented at an ED with symptoms of upper GI bleeding. All adult patients (aged older than 20 years) with a history of cirrhosis who presented at the ED of CGMH between 1 January 2001 and 31 December 2019 were included in the study. Upper GI bleeding was identified by two discharge diagnoses identified through International Classification of Diseases, Ninth Revision, Clinical Modification (ICD-9-CM) codes 578, 5780, 5789, 53501, 53541, 53160, 5342, 53140, 53141, 5310, 5317, 53120, 53121, 53551, 5307, 5312, 5316, 53401, 53400, 53421, 53420, 53531, 53441, 53440, 53461, 53460, 53100, and 53101 or International Classification of Diseases, Tenth Revision, Clinical Modification (ICD-10-CM) codes K920, K921, K922, K226, K256, K9421, K250, K252, K254, K286, K284, K282, K280, K274, K279, and I8501. Cirrhosis was identified through ICD-9-CM codes 571, 5712, 5715, and 5716 and ICD-10-CM codes K703, K7030, K7031, K74, K7460, K744, K745, K746, K7469, K743, K717, and P7881. Patients meeting the following criteria were excluded from the study: under 20 years of age; being a patient of trauma, obstetrics, or gynecology; and having no history of cirrhosis. Data on demographics, triage vital signs, blood tests, physical examinations, and treatment, including blood transfusion and medication, were collected. The MELD score was calculated for all patients [11].

### 2.2. Data Source and Preprocessing

EHR data were obtained from the Chang Gung Research Database, which is the largest multi-institutional EHR database in Taiwan and includes data from all seven CGMH facilities [12]. The study protocol was approved by the Institutional Review Board of CGMH (IRB no. 202200332B0). All patient information used in the study was anonymized and stripped of any identifying details. In terms of physiological measurements (e.g., vital signs), we replaced abnormally high or low values with missing values because they presumably indicated human error rather than actual measurements (e.g., weight > 400 kg). For continuous variables, we defined outliers as values exceeding 1.5 times the interquartile range of the empirical distribution. We replaced these values with missing values for later data imputation. For discrete variables, missing values were categorized as a new class, and categories with fewer than five occurrences were excluded. This ensured that enough samples were available for training, validation, and testing of the models. We used the Chi-squared test for categorical variables and Welch Two Sample *t*-test for continuous variables.

The proportion of missing data was high (>10%) for seven variables: blood urea nitrogen (BUN), basophil, eosinophil, lymphocyte, monocyte, segment, and blood glucose levels (missing rate ~15%). To solve this problem, we conducted a SenMice imputation, which yielded similar findings to those of the median imputation. Therefore, we adopted the results of median imputation in this study. After partitioning the data into training and testing data sets at a ratio of 90:10, we stored the testing set for evaluation once the final model was complete.

### 2.3. Prediction Model Construction and Evaluation

The ability of three classic ML models—the linear regression (LR) model, the XGBoost (XGB) model, and a three-layer neural network (NN) model—to predict mortality in patients with cirrhosis and upper GI bleeding was evaluated. To construct these models and select the optimal model through 10-fold cross-validation, we used the mljar-supervised AutoML tool and performed hyperparameter tuning [13]. The area under the receiver operating characteristic curve (AUROC) was selected as the evaluation metric. We used the optimal predictive model to examine the risk factors influencing the prognosis in follow-up groups, through Shapley Additive Explanations (SHAP) values. All statistical analyses were conducted using the R tableone package (version 0.12.0) and Python sklearn package (version 1.2.2).

## 3. Results

**Figure 1:**
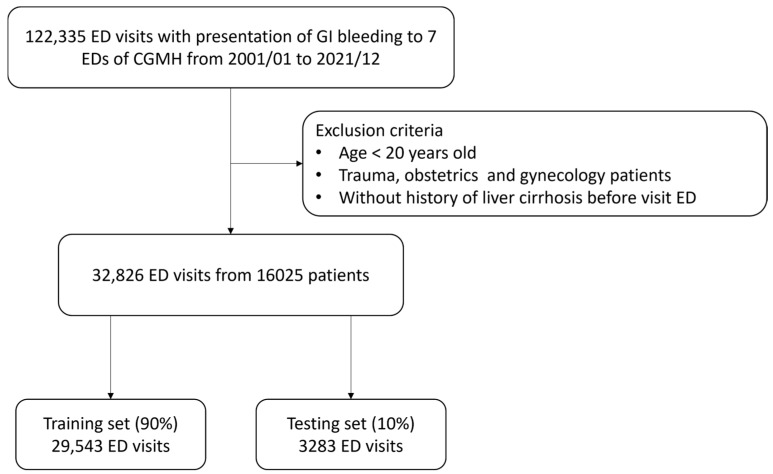
Flowchart of study population selection and division of the training and testing sets.

A total of 122,335 patients with GI bleeding were included in this retrospective study. After 89,509 patients had been excluded, 32,826 ED visits for upper GI bleeding were recorded for 16,025 patients with cirrhosis. As shown in Figure 1, the data were divided into a training set (90%) and a testing set (10%). Table 1 presents the characteristics of the in-hospital mortality, ED mortality, and survival groups. The overall rates of in-hospital mortality and ED mortality were 11.2% and 2.2%, respectively.

Compared with the survival group, the in-hospital mortality group exhibited more severe upper GI bleeding (indicated by triage level; 58.1% vs. 38.0%, *p* < 0.001) and were more likely to have been transported by an ambulance (11.5% vs. 5.4%, *p* < 0.001). The in-hospital mortality group also had a higher heart rate (99 vs. 96 beats per minute, *p* < 0.001) and lower systolic blood pressure (SBP; 112 vs. 126 mmHg, *p* < 0.001) at the ED. In terms of laboratory data, the in-hospital mortality group exhibited a lower hemoglobin level (8.83 vs. 9.27 g/dL, *p* < 0.001), a higher white blood cell (WBC) count (11.5 × 10^3^/μL vs. 7.71 × 10^3^/μL, *p* < 0.001), and a higher creatinine level (2.35 vs. 1.62 mg/dL, *p* < 0.001) compared with the survival group. The in-hospital mortality group had poorer liver function, including a higher international normalized ratio (INR; 1.74 vs. 1.35, *p* < 0.001), a higher aspartate aminotransferase level (307.6 vs. 92.0 U/L, *p* < 0.001), a lower albumin level (2.6 vs. 3.1 g/dL, *p* < 0.001), and a higher bilirubin level (6.5 vs. 2.6 mg/dL, *p* < 0.001). Compared with the survival group, the in-hospital mortality group received a higher MELD score on average (25 vs. 15, *p* < 0.001). Similar significant differences were found in the results of comparison between the ED death and ED survival groups.

**Figure 2:**
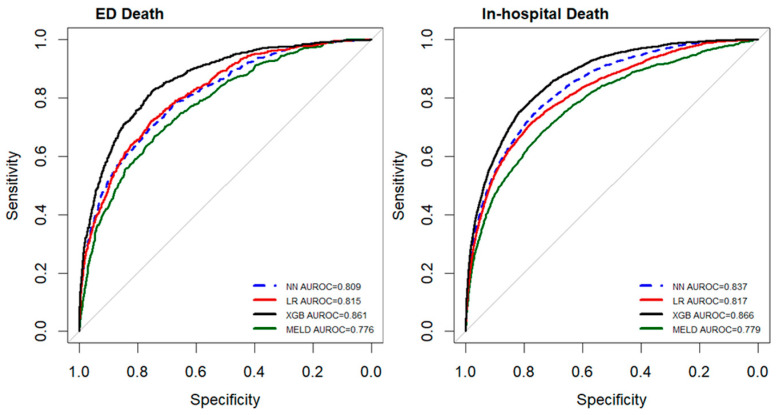
Comparison of the performance (AUROC) of various models in predicting mortality in patients with cirrhosis and upper GI bleeding. LR: logistic regression; XGB: XGBoost; NN: neural network; MELD: Model for End-stage Liver Disease.

Table 2 and Table 3 present the performance of the different models in predicting in-hospital mortality and ED mortality, respectively, in patients with cirrhosis and upper GI bleeding. The MELD score was selected as a traditional evaluation tool, and LR, XGB, and NN models were employed as ML models. The XGB model was the ML model with the highest predictive performance and was also found to perform better than the MELD score. As shown in Figure 2, the AUROCs for predicting in-hospital mortality, from high to low, were 0.866 for the XGB model, 0.837 for the NN model, 0.817 for the LR model, and 0.779 for the MELD. Similarly, the AUROCs for predicting ED mortality were 0.861 for the XGB model, 0.815 for the LR model, 0.809 for the NN model, and 0.776 for the MELD. Of the three ML models, the XGB model exhibited the highest accuracy (0.780), sensitivity (0.788), specificity (0.799), positive predictive value (0.309), and negative predictive value (0.967) in predicting in-hospital mortality. It exhibited a similar performance in the prediction of ED mortality.

**Figure 3:**
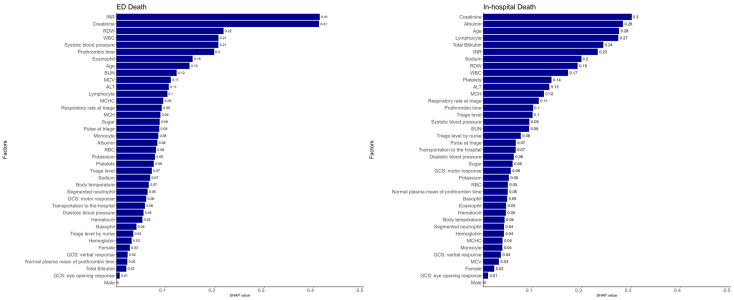
Ten most crucial features in the models.

**Figure 4:**
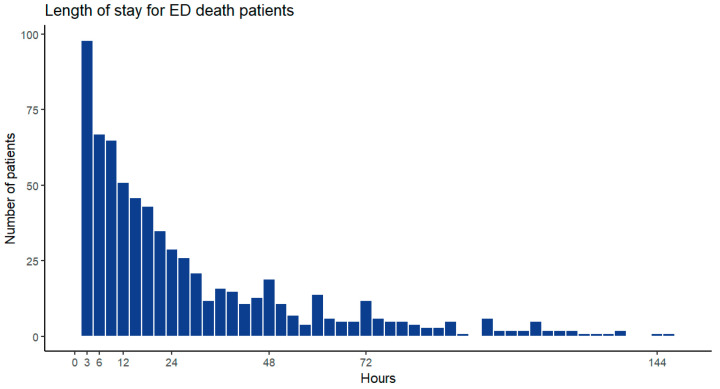
Length of stay of patients with ED death.

Figure 3 illustrates the SHAP values for the parameters used in predicting in-hospital mortality and ED mortality. The five parameters with the largest weights for predicting both in-hospital mortality and ED mortality were INR, creatinine level, red blood cell distribution width (RDW), WBC count, and age. The most crucial five parameters for the prediction of in-hospital mortality were albumin level, lymphocyte count, total bilirubin level, sodium level, and platelet count. The most crucial five parameters for the prediction of ED mortality were SBP, prothrombin time, eosinophil count, BUN level, and erythrocyte mean corpuscular volume (MCV). As shown in Figure 4, the median length of ED stay for the ED mortality group was 17.54 h (7.16–40.01 h).

## 4. Discussion

Upper GI bleeding can be life-threatening. Upper GI bleeding is associated with a 30-day mortality rate of 10%, and esophageal variceal bleeding is associated with a 30-day mortality rate of 50% [14]. Therefore, predicting the risk of mortality in patients with upper GI bleeding is essential in EDs, and ML models can be assistive in these predictions. In this study, we discovered a significant incidence of upper GI bleeding complicating cirrhosis, with an associated high mortality rate of 11.2%. We developed a ML model to predict mortality in patients with cirrhosis who present with upper GI bleeding at an ED, and the model was found to achieve outstanding performance.

Compared with traditional scoring systems, ML models make better predictions because they include a wider range of features and utilize more complex computational models. Previous studies have compared the performance of the Child–Pugh score and MELD score in predicting in-hospital mortality for cirrhotic patients with acute upper GI bleeding, reporting AUCs of 0.81 and 0.79, respectively [15]. We further compared the predictive abilities of traditional cirrhosis scoring tools with those of ML methods. Although scholars have attempted to use artificial neural networks to predict mortality in patients with cirrhosis and upper GI bleeding, these studies have primarily targeted long-term mortality rates, which are not applicable in the prediction of mortality at ED and during short-term hospital stays [16,17]. Hence, developing novel ML models for the early prediction of mortality in high-risk patients is essential for physicians to reduce the likelihood of adverse events by monitoring and treating patients both carefully and aggressively.

In this study, certain characteristics—INR, renal function, RDW, age, and WBC count—were identified by ML models as being the strongest predictors of both ED mortality and in-hospital mortality. As expected, higher INR was found to correlate with mortality in patients with cirrhosis and upper GI bleeding upon ED admission, consistent with previous research [18]. A higher INR indicates more severe bleeding resulting from poorer coagulation, reflecting poorer liver conditions in patients with cirrhosis. Controlling the patient’s coagulopathy is crucial and must be carried out promptly. Decreased renal function is a key predictor associated with reduced renal perfusion resulting from acute blood loss [19,20]. Older age is associated with higher mortality risk; in a previous study, Lecleire et al. discovered that being older than 60 years of age was a factor in predicting mortality in patients with upper GI bleeding, regardless of whether they had cirrhosis [5]. These indicate that the patient’s own health status (such as age and kidney function) is closely related to mortality. A large RDW suggests severe anemia or hemorrhage. Lee et al. reported that an RDW of 14.5% or greater was strongly linked to a high risk of upper GI bleeding [21]. The RDW is increased in patients with cirrhosis and positively correlates with coagulation, renal function, and bilirubin level [22]. A high WBC count was a predictor of short-term mortality in our study, presumably as a result of acute stress to hemorrhage. High WBC count can be observed in patients with GI bleeding, and the count reflects the severity of the bleeding [18,23,24]. When a patient presents with anemia, we should pay attention not only to hemoglobin levels but also to other hemogram indices.

Some other important features were found in our ML models to predict ED mortality. For instance, low SBP at triage suggests severe bleeding with hypovolemia and is a strong prognostic factor in cases of upper GI bleeding [25]. An elevated BUN level, common in patients with upper GI bleeding, is a result of blood protein breakdown and absorption in the upper GI tract; BUN values of 21 mg/dL or greater suggest severe upper GI bleeding [26]. A high MCV correlates with a high MELD score in patients with cirrhosis, and macrocytic anemia is closely linked to the degree of liver damage [27,28]. In patients with cirrhosis, a high MCV suggests poor liver function, which is associated with increased mortality risk.

Certain features were found to be more significant predictors of in-hospital mortality in our ML models. For instance, sodium, bilirubin, and albumin levels, which are typically used in traditional scoring systems such as the Child–Pugh score or MELD-Na score for cirrhosis, reflect cirrhotic status and correlate with mortality and morbidity in patients with cirrhosis. Hyponatremia develops secondary to portal hypertension, which is a consequence of advanced cirrhosis and is associated with high morbidity and mortality risks [29,30]. In patients with cirrhosis and upper GI bleeding, impaired liver function with high bilirubin and low albumin levels is associated with an increased mortality risk [18].

Most of our models exhibited high performance in predicting mortality in patients with cirrhosis and upper GI bleeding, for death either at the ED or in hospital. However, of our ML models, the NN model had relatively low predictive performance, presumably because our data were more suitable for traditional ML models or because our three-layer NN was not sufficiently complex. Compared with this NN model, the XGB model required less computational power and made its predictions more quickly, which would be beneficial for physicians in the ED, helping them prioritize examinations, treatment, and dispositions. Overall, establishing the optimal predictive model would assist healthcare providers in identifying patients at high risk of mortality, enabling them to administer more intensive treatments or initiate interventions earlier. Although our proposed ML model performed highly, further research is required to confirm its suitability for clinical applications.

This study has some limitations. First, we did not subgroup the patients with cirrhosis on the basis of the cirrhosis etiology, and we did not subdivide upper GI bleeding into esophageal, gastric, and duodenal categories. Since these patients arrive at the ED without having undergone an endoscopy, we do not know the source of their bleeding initially. Our goal is to provide mortality risk predictions early in the ED. Second, the times of death in the ED and wards overlapped, presumably because our wards were often at their full capacity, which resulted in a longer than necessary ED stay for some patients. Third, certain data points were missing, necessitating the use of values approximating the median for imputation. Fourth, some parameters that we did not check routinely at ED may affect the results of traditional scoring systems.

## 5. Conclusions

ML models can be used to predict mortality in patients with cirrhosis who present to an ED with upper GI bleeding, and their performance is higher than that of traditional scoring systems. Of all our ML models, the XGB model exhibits the highest predictive performance. INR, renal function, RDW, age, and WBC count are the strongest predictors in our ML models. The results generally align with our foundational knowledge and logic. We hope to utilize the convenience and speed of ML to predict the prognosis of patients shortly after their arrival in the emergency department. In addition to the overall results predicted by machine learning, we can also discuss expected treatment goals with patients based on factors such as age and chronic conditions (e.g., kidney disease). This approach allows us to determine whether to pursue aggressive treatment or consider the patient’s thoughts on a do-not-resuscitate (DNR) order, thereby enhancing the value of shared decision-making between physicians and patients. Although our ML models are applicable in this field, further research is required to determine their actual efficacy in clinical settings.

## Figures and Tables

**Table 1:** Comparison of the ED mortality, in-hospital mortality, and survival groups of patients with cirrhosis presenting with upper GI bleeding at an ED.

Demographics						
	In-Hospital Deaths	Survival		ED Deaths	Survival at ED	
	*n* = 3662	*n* = 29,164		*n* = 728	*n* = 32,098	
Age, years, mean (SD)	62.1 (13.9)	58.8 (13.9)	*p* < 0.001	59.1 (14.2)	59.9 (13.9)	*p* = 0.13
Gender, male, N (%)	2799 (76.43)	21,369 (73.27)	*p* < 0.001	583 (80.1)	23,585 (73.48)	*p* < 0.001
Arrival by ambulance, N (%)	420 (11.5)	1588 (5.4)	*p* < 0.001	99 (13.6)	1927 (6.6)	*p* < 0.001
Triage level 1 or 2, N (%)	2129 (58.1)	11,078 (38.0)	*p* < 0.001	471 (64.7)	12,727 (39.7)	*p* < 0.001
Triage vital signs						
BT, °C, median (IQR)	36.2 (36.0–36.8)	36.5 (36.0–36.9)	*p* < 0.001	36.2 (35.8–36.8)	36.4 (36.0–36.8)	*p* < 0.001
HR, bpm, median (IQR)	99 (84–114)	96 (82–112)	*p* = 0.006	102 (82–117)	97 (82–112)	*p* = 0.99
RR, breaths per minute, median (IQR)	19 (18–20)	20 (18–20)	*p* < 0.001	20 (18–22)	19 (18–20)	*p* = 0.04
SBP, mmHg, median (IQR)	112 (93–135)	126 (107–146)	*p* < 0.001	103 (82–128)	126 (106–146)	*p* < 0.001
DBP, mmHg, Median (IQR)	66 (54–78)	72 (61–84)	*p* < 0.001	60 (50–75)	72 (61–83)	*p* < 0.001
Initial laboratory data						
Hb, g/dL, mean (SD)	8.83, (2.47)	9.27, (2.68)	*p* < 0.001	8.61, (2.62)	9.24, (2.66)	*p* < 0.001
Hct, %, mean (SD)	26.5, (7.02)	28.1, (7.45)	*p* < 0.001	26.3, (7.48)	28.0, (7.42)	*p* < 0.001
WBC, 1000/uL, mean (SD)	11.5, (10.5)	7.71, (4.92)	*p* < 0.001	12.7, (10.4)	8.03, (5.6)	*p* < 0.001
Segment, %, mean, (SD)	77.1, (13.0)	70.9, (12.3)	*p* < 0.001	74.2, (15.9)	71.5, (12.4)	*p* < 0.001
Platelets, 1000/uL, mean (SD)	142.2, (103.4)	121.7, (84.2)	*p* < 0.001	134.8, (113.2)	123.7, (86.1)	*p* = 0.02
PT, seconds, mean (SD)	18.8, (10.0)	14.6, (5.40)	*p* < 0.001	21.6, (12.2)	14.9, (6.0)	*p* < 0.001
INR, mean (SD)	1.74, (1.0)	1.35, (0.52)	*p* < 0.001	2.0, (1.26)	1.4, (0.6)	*p* < 0.001
BUN, mean (SD)	46.9, (36.7)	31.1, (25.5)	*p* < 0.001	43.6, (35.2)	32.8, (27.3)	*p* < 0.001
Creatinine, mg/dL, mean (SD)	2.35, (2.01)	1.62, (1.86)	*p* < 0.001	2.47, (2.02)	1.68, (1.89)	*p* < 0.001
AST, U/L, mean (SD)	307.6, (1341.8)	92.0, (231.7)	*p* < 0.001	478.9, (2150.9)	113.9, (442.1)	*p* < 0.001
ALT, U/L, mean (SD)	94.6, (221.6)	46.2, (72.2)	*p* < 0.001	106.7, (285.1)	49.8, (89.7)	*p* < 0.001
Albumin, g/dL, mean (SD)	2.6, (0.6)	3.1, (0.7)	*p* < 0.001	2.5, (0.6)	3.0, (0.7)	*p* < 0.001
Total bilirubin, mg/dL, mean (SD)	6.5, (8.0)	2.6, (3.3)	*p* < 0.001	6.1, (7.1)	3.0, (4.3)	*p* < 0.001
Ammonia, ug/dL, mean (SD)	199.6, (218.9)	136.8, (104.9)	*p* < 0.001	286.9, (321.6)	143.2, (120.3)	*p* < 0.001
Na, mEq/L, mean (SD)	133.7, (6.84)	136.5, (4.85)	*p* < 0.001	135.2, (7.47)	136.4, (5.12)	*p* = 0.002
K, mEq/L, mean (SD)	4.6, (1.1)	4.1, (0.8)	*p* < 0.001	4.7, (1.4)	4.2, (0.8)	*p* < 0.001
MELD score, median (IQR)	25 (19–30)	15 (10–21)	*p* < 0.001	25, (19–31)	17 (11–22)	*p* < 0.001

ED: emergency department; BT: body temperature; SBP: systolic blood pressure; DBP: diastolic blood pressure; RR: respiratory rate; HR: heart rate; bpm: beats per minute; IQR: interquartile range; SD: standard deviation; Hb: hemoglobin; Hct: hematocrit; WBC: white blood cell; PT: prothrombin time; BUN: blood urea nitrogen; AST: aspartate aminotransferase; ALT: alanine transaminase; INR: international normalized ratio; MELD: Model for End-stage Liver Disease.

**Table 2:** Model performance for predicting in-hospital mortality in patients with cirrhosis and GI bleeding.

Model	AUC	Best Threshold	Accuracy	Sensitivity	Specificity	PPV	NPV
LR	0.817	0.112	0.765 (0.760–0.769)	0.717	0.771	0.281	0.956
XGB	0.866	0.101	0.780 (0.776–0.785)	0.788	0.779	0.309	0.967
NN	0.837	0.101	0.753 (0.748–0.756)	0.761	0.752	0.278	0.962
MELD score	0.779	0.137	0.725 (0.719–0.731)	0.687	0.731	0.282	0.938

LR: logistic regression; XGB: XGBoost; NN: neural network; PPV: positive predictive value; NPV: negative predictive value; MELD: Model for End-stage Liver Disease.

**Table 3:** Model performance for predicting ED mortality in patients with cirrhosis and GI bleeding.

Model	AUC	Best Threshold	Accuracy	Sensitivity	Specificity	PPV	NPV
LR	0.815	0.021	0.747 (0.743–0.752)	0.722	0.747	0.061	0.992
XGB	0.861	0.018	0.767 (0.762–0.771)	0.8	0.766	0.072	0.994
NN	0.809	0.019	0.757 (0.746–0.755)	0.701	0.752	0.06	0.991
MELD score	0.776	0.022	0.740 (0.734–0.746)	0.667	0.741	0.056	0.99

LR: logistic regression; XGB: XGBoost; NN: neural network; PPV: positive predictive value; NPV: negative predictive value; MELD: Model for End-stage Liver Disease.

## Data Availability

The data used in this study were obtained from the Chang Gung Research Database. All analyses were conducted in an isolated space. Data regarding the analytical process and original results are available from the corresponding author upon reasonable request.

## Abbreviations

GI	Gastrointestinal
MELD	Model for End-stage Liver Disease
ML	Machine learning
ED	Emergency department
LR	Linear regression
XGB	XGBoost
NN	Neural network
BUN	Blood urea nitrogen
SBP	Systolic blood pressure
WBC	White blood cell
INR	International normalized ratio
RDW	Red blood cell distribution width
MCV	Mean corpuscular volume
